# Commentary: QRS complex configurations in 12-lead electrocardiograms of dogs with monomorphic ventricular tachycardia or complete bundle branch block

**DOI:** 10.3389/fvets.2026.1877574

**Published:** 2026-07-29

**Authors:** Dragutin Novosel, Petar Žuljević, Tina Bečić

**Affiliations:** 1 Private Practitioner, Basel, Switzerland; 2 Department of Physiology, University of Split School of Medicine (MEFST), Split, Croatia; 3 Department of Cardiovascular Diseases, University Hospital of Split, Split, Croatia

**Keywords:** electrocardiography, electrical heart axis, ECG measurement, lead system, measurement error, reproducibility, data quality, Novosel formula

We read with great interest the recent article by Perego et al. (1) investigating QRS complex configurations in canine electrocardiograms. In the Materials and Methods section, the authors described the calculation of the mean electrical axis in the frontal plane (MEA) as follows: [(arctan I_amp_, aVF_amp_) × 180/π] (1). However, this formula was unclear to us and does not appear to correspond to the equation we originally published (2), to which the authors referred (1): ± arctan (2 × aVF/√3 × I). Our Equation 2 accounts for the modification required when the MEA is calculated using a combination of limb leads (I, II, III) and augmented leads (aVR, aVL, and aVF). The mathematical background of this correction (2/√3) has been explained elsewhere (3–5). Clarifying its use ensures methodological accuracy, reproducibility, and statistical consistency in further cardiology research, regardless of the subject studied (1–5).

While this distinction does not necessarily affect the conclusions of the article (1), it remains an important methodological point—not only for a single study, but also for study designs in which results are compared across studies or included in meta-analyses. It is therefore noteworthy that, in a recently published article by the same research group (6), the equation used accounts for the difference that arises when the MEA is calculated using a combination of limb and augmented leads.

Because the equation in article (1) was unclear to us, we requested and received the raw data used in this study (1), in accordance with the Data Availability Statement. The methods of data acquisition and the sophisticated data analysis are well-described (1).

However, here we comment only on the method used to calculate the MEA from the provided data, and explicitly not on the interpretation of its use in the study. As reported (1), the study design included measurements of ECG values from three consecutive heartbeats. As our focus is not the clinical value but only the methodology used to estimate the MEA, we used, for the purpose of our comment, the values from the first heartbeat of all 103 included subjects.

The authors (1) checked whether the data followed a normal distribution, which was not the case, and performed additional transformations and analyses.

Our goal is different and limited: we simply aimed to estimate how the MEA was calculated. Following the published analysis (1), we assumed that the distribution of the results did not follow a normal distribution. Our brief analysis of the originally reported MEA data confirmed this. We analyzed the distribution of the calculated MEA values using the Shapiro-Wilk test. The test indicated a significant deviation from normality (W = 0.7944, *p* < 0.01). Therefore, non-parametric statistical methods were used.

For our purpose, we used the I and aVF lead values from the first QRS complex of all 103 subjects and compared the reported MEA from the raw data with two of our own calculations, one of which we assumed to be incorrect and the other of which we considered correct. We rounded our results to two decimal places, consistent with the reporting in the original study (Table 1).

**Table 1:** Descriptive statistics for the mean electrical axis (MEA) calculated using three different approaches/Equations:1) Originally reported arctangent function based on Iamp and aVFamp, 2) the equation arctan (aVF/I) × 180/n and 3) the corrected equation arctan (2 × aVF/(√3 × I)) × 180/n.

Descriptive statistics	Originally reported equation (arctan Iamp, aVFamp) × 180/π	Wrong equation (arctan (aVF/I) × 180/π)	Corrected equation (arctan (2 × aVF/(√3 × I)) × 180/π)
N	103	103	103
Mean	−1.04	−0.74	0.15
SEM	9.29	9.30	9.26
Median	68.03	68.03	70.74
Min.	−163.69	−163.69	−161.33
Max.	165.96	165.96	163.90
Q1	−98.22	−98.20	−97.13
Q3	82.08	83.81	84.64

Statistical analysis: *Post-hoc* pairwise Wilcoxon signed-rank tests were performed following the Friedman test. *P*-values were adjusted for multiple comparisons using the Holm-Bonferroni method. A Holm-Bonferroni-adjusted *p*-value < 0.01 was considered statistically significant.

The estimated differences between the original results [column 1: arctan (I_amp_, aVF_amp_) × 180/π] and the results calculated using the formula without correction [column 2: arctan (aVF/I) × 180/π] were not statistically significant (*p* = 1.00). Noteworthy is that, out of 103 results, only in 5 cases were the results not equal. The originally estimated MEA (column 1) and those calculated using the equation without correction (column 2) both differed from the values calculated using the equation including the modification factor [column 3: arctan (2 × aVF / √3 × I) × 180/π]; for both comparisons, *p* < 0.001.

The differences between all three MEA values depend on the data set and were minimal in the present data. However, the difference between the MEA values calculated with correction and those calculated without correction is not a linear function. It ranges from zero (at MEA values of 0, 180, and ±90 degrees) up to 4.12 degrees. For example, the MEA calculated with correction is 47.06 degrees, whereas the value calculated without correction is 42.94 degrees, corresponding to a difference of 4.12 degrees or 9.59%. Please note that this percentage refers to the maximal absolute difference expressed in degrees, and not to the maximum relative percentage difference. The relative difference reaches its maximum near an axis of zero degrees, where it exceeds 15%. Calculations can be provided if necessary. Although this avoidable difference of more than 9% will rarely be clinically relevant in daily practice, it may be significant in big data analyses or epidemiological studies.

Based on our analysis, we conclude that the original study (1) the reported MEA values are numerically consistent with calculation without the correction factor and not with calculation using the corrected equation for the leads I and aVF.

As stated above, our comment does not in any way diminish the value of the original study; it simply aims to clarify the calculation of the MEA.
